# Superior mesenteric arteriovenous fistula embolization with a vascular plug for upper gastrointestinal hemorrhage: a case report

**DOI:** 10.1186/s42155-022-00296-0

**Published:** 2022-07-22

**Authors:** Austin Shinagawa, Zaeem Billah, Kartik Kansagra, Cuong Lam, Geogy Vatakencherry

**Affiliations:** Kaiser Permanente Los Angeles Medical Center, Vascular and Interventional Radiology, 4867 Sunset Boulevard, Los Angeles, CA 90027 USA

**Keywords:** SMA, SMV, mesenteric, fistula, embolization

## Abstract

**Background:**

Superior mesenteric arteriovenous fistula is a rare vascular anomaly often presenting with sequelae of portal hypertension, heart failure, or mesenteric ischemia. This report describes a patient with a previously unidentified superior mesenteric arteriovenous fistula who presented with variceal bleeding, thought to be the leading cause of mortality associated with this condition. Although this patient was initially referred for a transjugular intrahepatic portosystemic shunt procedure, following a thorough review of her clinical history and imaging, she instead underwent embolization of the arteriovenous fistula likely responsible for her symptoms.

**Case Presentation:**

A 75-year-old woman with a past surgical history of extensive small bowel resection presented with active variceal bleeding requiring transfusions. She was referred to vascular and interventional radiology for transjugular intrahepatic portosystemic shunt procedure; however, her clinical presentation was inconsistent with cirrhosis. This prompted a further review of her imaging, which identified a superior mesenteric arteriovenous fistula as the probable etiology of her varices. This fistula was subsequently embolized with a vascular plug and follow-up upper endoscopy at 1-month demonstrated complete resolution of her varices.

**Conclusions:**

This report highlights a potential etiology of variceal bleeding in the acutely ill patient. Through a thorough consultation, the patient described here was able to avoid a procedure with the potential to cause catastrophic consequences, and instead receive the appropriate treatment for an uncommon condition.

**Level of Evidence:**

Level 4, Case Report.

## Background

The primary etiologies of superior mesenteric arteriovenous fistulas (SMAVF) are traumatic, iatrogenic, and congenital (Shintani et al., 2011; Wu et al., 2008; Rimon et al., 2008). This rare vascular anomaly often presents with sequelae of portal hypertension, heart failure, or mesenteric ischemia (Kato et al., 1993). Although widely variable, common signs and symptoms of SMAVF include abdominal pain, diarrhea, upper or lower gastrointestinal bleeding, ascites, and anemia (Shintani et al., 2011; Wu et al., 2008; Rimon et al., 2008; Kato et al., 1993; An et al., 2013; Zhao et al., 2018; Landi et al., 2015; Huang et al., 2018; Jargiełło et al., 2019; Zhao et al., 2014; Farshidmehr et al., 2019). The most consistent specific physical exam finding is an abdominal bruit (Kato et al., 1993).

Variceal bleeding is suspected to be the leading cause of mortality associated with SMAVF (Jargiełło et al., 2019). If a SMAVF is not initially identified, such patients presenting with acute gastrointestinal bleeding might be referred to vascular and interventional radiology (VIR) for a transjugular intrahepatic portosystemic shunt (TIPS) procedure. However, a thorough consultation can preclude the potentially catastrophic consequences of creating a portosystemic shunt in the presence of an arteriovenous fistula (An et al., 2013). The present case report describes a patient presenting with gastrointestinal hemorrhage initially referred to VIR for a TIPS procedure, in whom a SMAVF was discovered and subsequently embolized.

## Case Presentation

A 75-year-old woman presented to an outside hospital with hematochezia and hematemesis. She had a surgical history of two small bowel resections four and six years prior to presentation. Following these resections, she was left with 35 centimeters of small bowel and developed short-gut syndrome reliant on total parenteral nutrition (TPN). She had also developed ascites within the preceding four years, presumed to be secondary to parenteral nutrition-associated liver disease.

The patient was resuscitated with seven units of packed red blood cells and four units of fresh frozen plasma. Upper endoscopy visualized grade 3 esophageal and grade 2 gastric varices. Balloon tamponade was performed with a Linton tube and the patient was transferred to a tertiary care hospital on octreotide and a proton pump inhibitor. VIR was consulted to evaluate the patient for a TIPS procedure; however, several findings were inconsistent with a working diagnosis of portal hypertension due to cirrhosis.

**Fig. 1:**
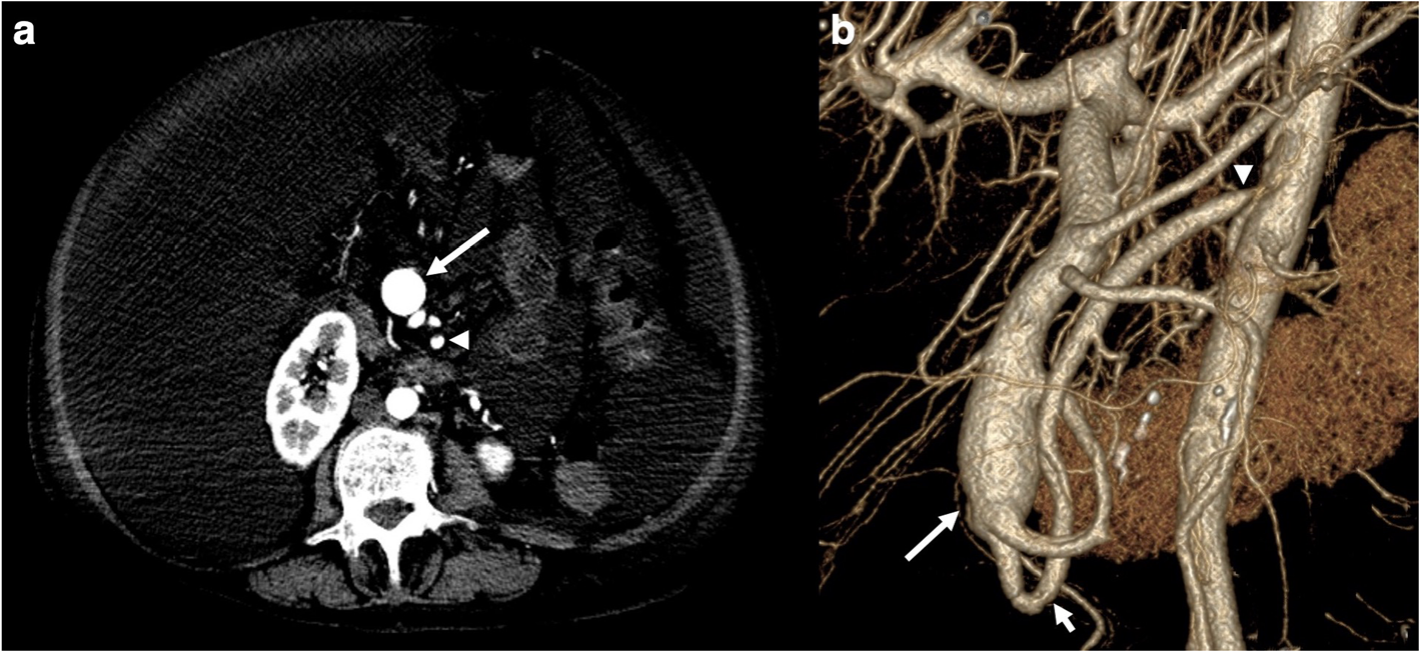
Contrast-enhanced abdominal CT scan (arterial phase) of a superior mesenteric arteriovenous fistula (SMAVF) (**a**) Axial image shows a SMAVF (arrowhead) feeding into a dilated superior mesenteric vein (arrow) (**b**) 3D reconstruction shows the SMAVF (short arrow) connecting the superior mesenteric artery (arrowhead) to an ectasia of the superior mesenteric vein (long arrow)

The patient did not have typical cirrhotic risk factors (no hepatitis, alcohol use disorder, or obesity) and fibrosis was not seen on a liver biopsy 4 years prior, so long-term use of TPN was the primary factor that would predispose her to cirrhosis. These considerations, along with a lack of cirrhotic features on an abdominal computed tomography angiography (CTA) scan, indicated that cirrhotic liver disease was unlikely. Closer inspection of the abdominal CTA scan revealed abnormal hyperenhancement and dilation of both the superior mesenteric vein (SMV) and portal vein in the arterial phase. Further review confirmed the presence of a SMAVF distal to the intestinal branches of the superior mesenteric artery (SMA) (Fig. 1).

**Fig. 2:**
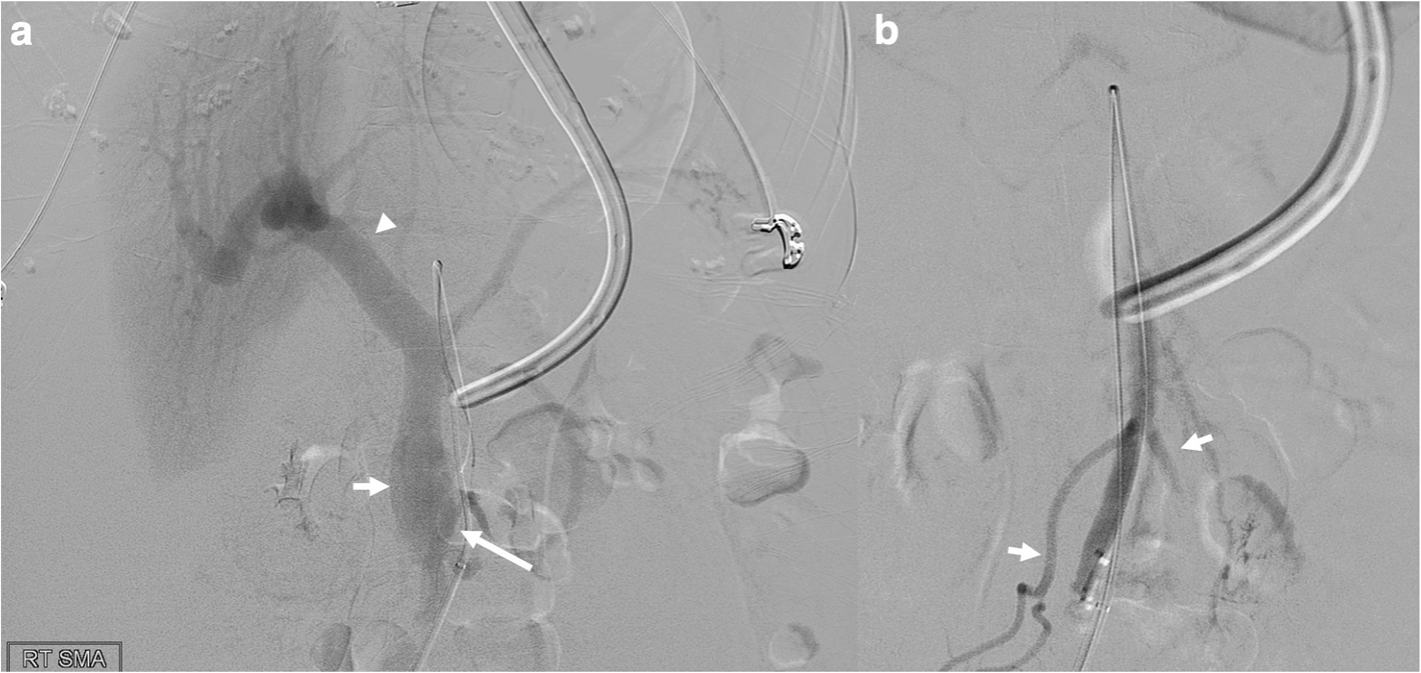
Pre- and post-embolization angiography of superior mesenteric artery (**a**) Digital subtraction angiography (DSA) prior to embolization demonstrates superior mesenteric arteriovenous fistula (long arrow) draining into dilated superior mesenteric vein (short arrow) and portal vein (arrowhead) (**b**) DSA following embolization with AMPLATZER Vascular Plug II (AGA Medical, Golden Valley, Minnesota) shows complete occlusion of fistula with preservation of major superior mesenteric artery intestinal branches (short arrows)

We proceeded with embolization of the SMAVF to reduce portal pressure. A 6-F 25 cm sheath was placed in the right common femoral artery. A 5-F Cobra 1 catheter (Merit Medical, South Jordan, Utah) and 2.4-F Progreat microcatheter (Terumo, Tokyo, Japan) were used to cannulate the SMV across the fistula from the SMA (Fig. 2a). Then a 9 mm Eclipse dual-lumen balloon occlusion catheter (Cobalt Medical, Wayne, New Jersey) was used to measure pressure in the distal SMA (9.3 kPa) and the wedge pressure of the SMV (7.1 kPa). The balloon catheter was then exchanged for a 4-F angled Glidecath (Terumo, Tokyo, Japan) to accommodate an 0.035” Rosen wire (Cook Medical, Bloomington, Indiana). Then, a 4-F 90 cm Flexor sheath (Cook Medical, Bloomington, Indiana) was advanced into the peripheral aspect of the SMV outflow and an 8-mm AMPLATZER Vascular Plug II (AGA Medical, Golden Valley) was deployed, extending from the SMV outflow into the distal SMA inflow. Post-embolization angiogram ensured that the plug was distal to major arterial intestinal branches and demonstrated an absence of portal venous filling (Fig. 2b). The patient was transferred to the intensive care unit and subsequently discharged in stable condition on postprocedural day 8. Although her ascites persisted, she had no further bleeding during the remainder of her hospitalization.

An upper endoscopy at 1 month post-procedurally demonstrated complete resolution of both esophageal and gastric varices. Liver elastography performed shortly after this visit revealed minimal scarring with a kPa of 9.1 (METAVIR stage F2). At her 2, 3, 12, and 16-month postprocedural follow-up visits, the patient’s ascites had resolved (weaned off diuretics) and she denied any recurrence of gastrointestinal bleeding. Four-phase liver protocol computed tomography scan at 16-months post-procedurally demonstrated no further evidence of a portosystemic shunt, a patent portal venous system, resolution of ascites, and no recurrence of esophageal or gastric varices. This patient will continue to be followed in the VIR clinic indefinitely for surveillance of the SMAVF and liver disease management.

## Conclusions

The management of SMAVF is either surgical or endovascular. The surgical option provides definitive fistula closure, but is also associated with higher perioperative risk (Zhao et al., 2018). Endovascular repair is currently the prevailing option, particularly in patients with high surgical risk, prior abdominal surgeries, or emergent presentation (Zhao et al., 2014). It can be performed through a transarterial, transvenous, or combined approach, and typically involves covered stents or coil embolization for fistula closure (Shintani et al., 2011; Wu et al., 2008; Kato et al., 1993; An et al., 2013; Zhao et al., 2018; Landi et al., 2015; Jargiełło et al., 2019; Zhao et al., 2014; Farshidmehr et al., 2019; Howley et al., 2019; Yamaguchi et al., 2018; Wang et al., 2016). Vascular plugs, as described in the present case, also appear to be an effective option, with potentially less risk for migration into the portal venous system than coils (Rimon et al., 2008; Huang et al., 2018). Other potential complications of endovascular SMAVF closure are portal vein thrombosis and mesenteric ischemia (Landi et al., 2015).

If the patient had received a TIPS procedure as requested on the initial consult, she would not only be at significant risk for heart failure, but also recurrent variceal bleeding (An et al., 2013). Fortunately, by receiving a thorough preprocedural consultation, this patient was able to avoid an unnecessary or even harmful procedure and instead receive the appropriate treatment. Following embolization, she had clinical improvement as well as both endoscopic and radiographic evidence of variceal resolution, suggesting that the etiology of her symptoms was secondary to the SMAVF instead of cirrhosis. The mild hepatic scarring demonstrated on elastography was instead suspected to be due to chronic TPN; however, chronic portal hypertension was unlikely due to the rapid resolution of her varices.

In summary, the present case describes a successful SMAVF closure via vascular plug embolization, in a patient presenting with gastrointestinal hemorrhage initially referred to VIR for a TIPS procedure.

## Data Availability

Not applicable.

## Abbreviations

SMAVF: Superior mesenteric arteriovenous fistula
VIR: Vascular & interventional radiology
TIPS: Transjugular intrahepatic portosystemic shunt
TPN: Total parenteral nutrition
CTA: Computed tomography angiography
SMA: Superior mesenteric artery
SMV: Superior mesenteric vein

## References

[CR1] An T, Zhou S, Song J, Jiang T, Li X, Wang W (2013). Massive gastrointestinal bleeding secondary to superior mesenteric arteriovenous fistula. Am J Gastroenterol.

[CR2] Farshidmehr P, Zafarghandi MR, Sadat A, Sayarifard A (2019). Coil Embolization of an Iatrogenic Arteriovenous Fistula between the Superior Mesenteric Artery and Vein: A Case Report. J Tehran Heart Cent.

[CR3] Howley IW, Stein DM, Scalea TM (2019). Outcomes and complications for portal vein or superior mesenteric vein injury: No improvement in the era of damage control resuscitation. Injury..

[CR4] Huang IKH, Ameli-Renani S, Ratnam L, Morgan RA (2018). Embolization of a Mesenteric Arteriovenous Fistula. J Vasc Interv Radiol.

[CR5] Jargiełło T, Sobstyl J, Kasztelan-Szczerbińska B, Sojka M, Pyra K (2019). Endovascular treatment of the superior mesenteric arteriovenous fistula complicated by gastrointestinal bleeding. Turk J Gastroenterol.

[CR6] Kato S, Nakagawa T, Kobayashi H, Arai E (1993). Superior mesenteric arteriovenous fistula: Report of a case and review of the literature. Surg Today.

[CR7] Landi F, Ronot M, Abdel-Rehim M, Sibert A, Bissonnette J, Soubrane O (2015). Combined transhepatic portal venous and transarterial treatment of superior mesenteric arteriovenous fistula in a patient with cirrhosis. J Vasc Interv Radiol.

[CR8] Rimon U, Heldenberg E, Golan G, Shinfeld A, Garniek A (2008). Amplatzer Vascular Plug: Expanding the Applications Spectrum. Cardiovasc Intervent Radiol.

[CR9] Shintani T, Mitsuoka H, Masuda M (2011). Transcatheter coil embolization of an iatrogenic superior mesenteric arteriovenous fistula: report of a case. Surg Today.

[CR10] Wang C, Zhu X, Guo G-H, Shu X, Wang J, Zhu Y (2016). Superior mesenteric arteriovenous fistula presenting as gastrointestinal bleeding: case report and literature review. Rev Esp Enferm Dig.

[CR11] Wu C-G, Li Y-D, Li M-H (2008). Post-traumatic superior mesenteric arteriovenous fistula: endovascular treatment with a covered stent. J Vasc Surg.

[CR12] Yamaguchi H, Murata S, Onozawa S, Sugihara F, Saito H, Kumita S-I (2018). Coil embolization using microballoon assistance combined with the double-catheter technique for a large superior mesenteric arterial pseudoaneurysm and fistula secondary to acute pancreatitis. J Vasc Surg Cases Innov Tech.

[CR13] Zhao Y, Li Z, Zhang L, Wei B, Zeng X, Fu P (2014). Portal vein thrombosis secondary to embolization of superior mesenteric arteriovenous fistula. Ann Vasc Surg.

[CR14] Zhao Y, Xie B, Liu Q, Luo R, Wan Y, Malik K (2018). Endovascular Treatment of Post-traumatic Superior Mesenteric Arteriovenous Fistula: A Case Report. Ann Vasc Surg.

